# Sensitivity of assays for TSH-receptor antibodies

**DOI:** 10.1007/s40618-016-0520-y

**Published:** 2016-08-16

**Authors:** V. Bitcon, J. Donnelly, D. Kiaei

**Affiliations:** Siemens Healthcare Diagnostics Inc, Tarrytown, NY USA

To the Editor in Chief

We wish to share our concern with the scientific design, validity and results of the work by T. Diana, C. Wuster, M. Kanitz and G. J. Kahaly published in your journal (Highly variable sensitivity of five binding and two bioassays for TSH-receptor antibodies, J. Endocrinol. Invest. Published online 19 May 2016, doi:10.1007/s40618-016-0478-9). In this publication, the authors conclude that sensitivity is highly variable between binding and bioassays for TSH-receptor antibodies (TSHR-Abs). In fact, they incorrectly identified one of the commercial assays, the Siemens IMMULITE^®^ 2000 TSI assay, as a TSHR-binding inhibitory immunoglobulins (TBII) assay (in multiple instances throughout the manuscript). This is incorrect as the IMMULITE TSI assay has a patented, novel “bridge” format for the direct detection of thyroid stimulating autoantibodies to TSHR and does not rely on binding inhibition of TSH or any other ligand used in TBII assays. It also has a published independently derived 98.6 % sensitivity and 98.5 % specificity when tested with patients suffering from a variety of thyroid and autoimmune diseases including Hashimoto’s thyroiditis (HT) [1].

In their study, the authors tested various samples from patients with Graves’ disease (GD) and observed a 95 % sensitivity for the IMMULITE TSI assay which is close but lower than the published 98.6 % (95 % CI 96.8–99.5) sensitivity [1]. The authors tested 20 hypothyroid Hashimoto’s patients with a blocking bioassay and several binding assays and obtained positive results. This is expected since Hashimoto’s patients have in some cases both stimulating and blocking antibodies [2, 3]. It stands to reason that if the patient is hypothyroid, then blocking antibody avidity and/or titer would dominate irrespective of the presence of stimulating antibodies. There is a growing list of publications that show the prevalence of TSHR stimulating autoantibodies in HT patients using bioassays. Wall et al. [4] found that 22 % (4/17) HT patients without thyroid-associated orbitopathy (TAO) were TSI positive using Thyretain™ TSI Reporter BioAssay. Another clinical study using a different bioassay had similar conclusion and showed 7 % of the HT patients have stimulating autoantibodies [5]. A recent publication by the authors of the subject manuscript showed that 5.5 % of HT patients and 68.2 % of HT patients with TAO were TSI positive when tested with the Thyretain bioassay [6]. Astonishingly, the authors did not test the HT samples with the stimulating bioassay for this study and only tested them with the blocking bioassay. Therefore, we do not know the stimulating autoantibody status of the HT patients. Furthermore, the HT population selected appears to not be random as all tested positive with blocking bioassay. The prevalence of TSHR blocking autoantibodies in HT patients is reportedly 9 % [7]. The IMMULITE TSI assay has a published specificity of 96.4 % when 111 randomly selected HT patients were evaluated [1]. Independent clinical studies have confirmed the high performance of IMMULITE TSI assay showing 100 % sensitivity and 98 % specificity with GD and HT patients [8, 9]. The testing of the HT patients with the Thyretain bioassay may fail to detect all the stimulating antibodies due to its lower sensitivity (92 % according to Thyretain assay package insert) compared to the IMMULITE TSI assay with 98.6 % sensitivity. Stimulating antibody tests that do not rely on bioassay will detect stimulating antibodies even in the presence of blocking antibodies allowing the physician to monitor the natural history of the thyroiditis and anticipate the potential for latent development of Graves’ disease.

In addition to the misrepresentation of the IMMULITE TSI assay, we discovered several errors in the data analysis and the results. The authors report in Table 2 that *all* 20 HT patients tested positive with Roche’s assay. However, Table 4 shows that the results had a (min–max) range of 0.3–40 IU/L. Given the assay’s cut-off of 1.75 IU/L, it is clear that (at least) one HT sample (with reported dose of 0.3 IU/L) was negative in Roche’s assay, contrary to the data presented in Table 2. Secondly, Table 2 shows zero HT patients for both TSAb+ and TSAb− categories which is a contradiction in itself. If zero patients were TSAb + , then all 20 HT patients must have been TSAb- or vice versa. The same contradiction appears for the healthy controls and GD patients, zero healthy patients are TSAb+ and zero patients are TSAb−. Thirdly, the data presented in Table 2 mislead the reader into thinking TSAb tests were run and none of the HT patients were positive when in fact according to the text in Results section, HT patients were only tested with the TBAb (blocking) bioassay. Therefore, this study is incomplete and inconclusive as the authors neglected to test HT patients with the TSAb assay despite various publications, including the authors own previous work, showing the prevalence of TSHR stimulating autoantibodies in HT patients [6].
